# Dietary Interventions to Prevent Childhood Obesity: A Literature Review

**DOI:** 10.3390/nu13103447

**Published:** 2021-09-28

**Authors:** Ana Rita Pereira, Andreia Oliveira

**Affiliations:** 1 Faculty of Health Sciences (Nutrition Sciences), University Fernando Pessoa, Rua Carlos da Maia 296, 4200-150 Porto, Portugal; 35460@ufp.edu.pt; 2 EPIUnit—Instituto de Saúde Pública da Universidade do Porto (Institute of Public Health of the University of Porto), Rua das Taipas 135, 4050-600 Porto, Portugal; 3 Laboratory for Integrative and Translational Research in Population Health (ITR), Rua das Taipas 135, 4050-600 Porto, Portugal; 4 Department of Public Health and Forensic Sciences, and Medical Education, Faculty of Medicine, University of Porto, Alameda Professor Hernâni Monteiro, 4200-319 Porto, Portugal

**Keywords:** pediatric obesity, children, dietary interventions, diet, prevention

## Abstract

Several dietary interventions have been conducted to prevent/reduce childhood obesity, but most of them are known to have failed in tackling the obesity epidemic. This study aimed to review the existing literature on dietary interventions for the prevention of childhood obesity and their effectiveness. A literature search was conducted using PubMed Central^®^. Only articles published between 2009 and 2021, written in English, conducted in humans, and including children and/or adolescents (<18 years old) were considered. The majority of studies were school-based interventions, with some addressing the whole community, and including some interventions in the food sector (e.g., taxation of high fat/sugar foods, front-of-pack labelling) and through mass media (e.g., restrictions on food advertising for children) that directly or indirectly could help to manage childhood obesity. Most of the programs/interventions conducted focus mainly on person-based educational approaches, such as nutrition/diet education sessions, allied to the promotion of physical activity and lifestyles to students, parents, and school staff, and less on environmental changes to offer healthier food choices. Only a few trials have focused on capacity building and macro-policy changes, such as the adaptation of the built environment of the school, serving smaller portion sizes, and increasing the availability and accessibility of healthy foods and water in schools, and restricting the access to vending machines, for example. Overall, most of the intervention studies showed no consistent effects on changing the body mass index of children; they have only reported small weight reductions, clinically irrelevant, or no effects at all. Little is known about the sustainability of interventions over time.

## 1. Introduction

In the recent past, there was a shift from prevailing infectious diseases to a high prevalence of chronic and degenerative diseases associated with lifestyle choices [1]. Obesity is one of the conditions that has dramatically increased all over the world, and children, in particular, are a cause of public health concern [2].

The prevalence of overweight and obesity has increased substantially over the past four decades, and an epidemiological transition from underweight to overweight and obesity has been described throughout the world [3]. This alarming rise has been observed in all regions, including developing countries, with an increase of overweight and obesity prevalence from 1980 to 2013 of 8.1% to 12.9% (in boys) and 8.4% to 13.4% (in girls) [2]. These increases have been also reported in developed countries, among children and adolescents, with 23.8% of boys and 22.6% of girls having either overweight or obesity in 2013 [2]. Although the prevalence is clearly higher in developed countries at all ages, the differences between sexes are small. Nonetheless, the prevalence of childhood obesity in the United States and some European countries has apparently reached a plateau [4], but it continues at high rates.

Obesity is a complex, multifactorial disease. Although genetics may be an important etiological factor for obesity development, genes do not fully explain the huge and fast increase of obesity at the population level [4,5]. It is believed that this obesity epidemic may be due to gene–environment interactions [6], enhanced by an increasingly permissive obesogenic environment, with different levels of determinants [1,7]. There are micro-environmental settings, such as schools, workplaces, homes, and neighborhoods, and these are influenced by macro-environmental sectors, such as the health system and the food industry that may be key settings to tackle the obesity epidemic [7]. Now, more than ever, individuals are embedded in a more permissive environment with concern to eating habits and are more likely to adopt sedentary behaviors. It has been recognized many years ago in the Ottawa Charter that it is very important to promote supportive environments [8]. In the case of children, the family and school are included in a wider proximal context [7].

It is well known that diet and other habits are shaped at the earlier stages of life and maintained through adulthood [9]. With the current increasing rates of childhood obesity, there has been a growing amount of research focusing on the determinants of obesity in children and their families, and several studies have described possible dietary/nutritional interventions to prevent childhood obesity. It is known that interventions that are mostly based on educational, behavioral, or pharmacological measures are not very effective in preventing and treating obesity [10,11].

This study aims to review the existing literature on dietary interventions for the prevention of childhood obesity and to assess their effectiveness.

## 2. Materials and Methods

A literature search was conducted using the PubMed Central^®^ search engine, the most comprehensive dataset for biomedical literature. The search expression used for this search included the mesh terms “(pediatric obesity) OR (childhood obesity) AND (primary prevention) AND “diet”. Due to the extensive amount of published data, we limited the timeline to have only articles from 2009 up to 2019. An update search was then performed to include studies published between 2019 and 2021. Only articles written in English, conducted in humans, and including children and/or adolescents (<18 years old) were included. This search yielded 538 articles, of which we excluded 513, including 25 in this study. The literature search had three stages, the search for the titles, then abstracts, and finally the full-text papers were searched and retrieved (when deemed of interest). Some articles were discarded because they did not report measures to prevent obesity in children (n = 192) or because these measures were implemented only in adults (n = 321). 

**Figure 1:**
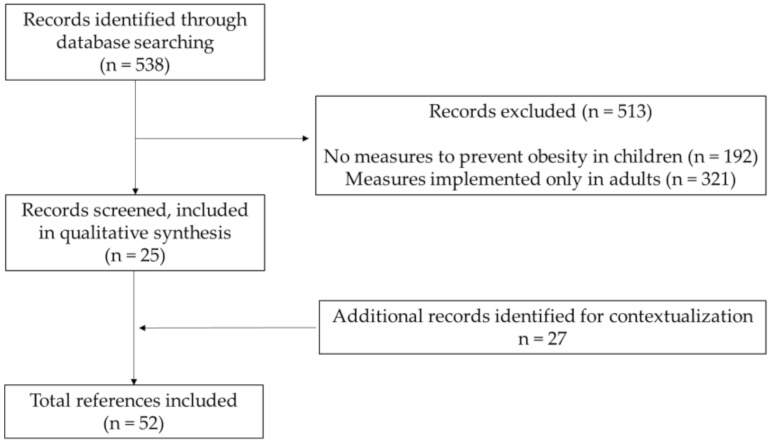
Flowchart of studies’ selection.

Additional papers (n = 27) were included in this review from a snowball process or searched to put into context the dietary interventions for the prevention of childhood obesity, totaling 52 references. Figure 1 presents the flowchart of the studies’ selection.

In this literature review, dietary interventions to prevent childhood obesity were grouped and described into four levels: school-based interventions, community-based interventions, interventions through mass media, and food sector interventions.

## 3. Results

To prevent obesity in children there is a need to take multidimensional actions at different levels, including the individual, familial, institutional, and environmental levels. At the moment, these types of multilevel interventions seem to be the most promising ones to actually prevent/manage obesity. In particular, children are very influenced by social and environmental conditions, so at these ages, community-based interventions, changing the supportive environment, seem to play an especially important role [12].

Table 1 provides a descriptive summary of the dietary interventions to tackle childhood obesity, described in detail below.

### 3.1. School-Based Interventions

The Ballabeina study is a cluster-randomized controlled single-blinded trial that took place in some preschools in Switzerland, designed to study the effect of a multidimensional lifestyle intervention on aerobic fitness and adiposity, mainly in migrant preschoolers with the duration of over one school year [13]. This study included 652 preschool children with a mean age of 5.1 years. The interventions comprised a physical activity program, lessons on nutrition, media use, and sleep, and adaptation of the built environment of the preschool. The dietary intervention included weekly nutrition lessons given by a dietician; the students could learn about balanced nutrition and healthy nutritional behaviors in a didactic way. These lessons were centered on five messages: “drink water”, “eat fruit and vegetables”, “eat regularly”, “make clever choices”, and “turn your screen off when you eat”, which were developed in collaboration with the Swiss Society for Nutrition. These messages were also described on funny cards that children could get with a task to implement the message at home. After 4 months of intervention, the results showed no differences between the groups in the children’s body mass index (BMI). However, an increase in aerobic fitness by the end of the intervention was reported, and children in the intervention group also showed beneficial effects in the percentage of body fat (−1.1%), and their motor agility, when compared with the children in the control group. It was also possible to observe benefits in reported physical activity, media use (less screen time in boys), and eating habits, such as an increase in fruit and vegetable consumption in the intervention group [13]. 

In the Netherlands, a school-based trial was implemented including students from the ages of 12–14 years old (n = 1108), within a multidimensional health promotion intervention [14,15]. There were 10 intervention and eight control secondary schools included. The intervention included an educational component, with classes in biology and physical education, and a computer-based information program; and an environmental component, with propositions such as serving smaller portion sizes in the canteen and healthier food options, or restricting the access to vending machines. There were also posters affixed to create more awareness about which foods were healthier and which were not. With a twenty-month follow-up, it was observed in the intervention group a reduction in body composition measures, such as skinfold thickness, lower consumption of sugar-containing beverages at 12 months, and less screen time (but only in boys) [14,15].

A school-based obesity-prevention trial in Chile evaluated the effect of weekly physical activity classes and classes on healthy nutrition for parents and students from 1st to 8th grade; 2141 schools were in the intervention group and 945 in the control group [16]. Some environmental changes were also made, including instructing school kiosks to offer healthier options to students and still remain lucrative. The results showed a reduction in BMI z-scores in boys after 6 months of intervention and better physical fitness in both genders. On the other hand, the modifications in the kiosk’s food availability did not seem to change the students’ food choices [16].

The school-based Healthier Options for Public Schoolchildren (HOPS) is a randomized trial implemented over two school years (2004–2005 and 2005–2006) that included six elementary schools (4588 children aged 6 to 13 years; 48% Hispanic) in Osceola, Florida. Interventions implemented included modifications in the school menu, school gardens, and physical activity [17]. Complementarily, there were healthy nutrition and physical activity lessons for the students and parents through monthly newsletters. After 2 years, it was possible to observe a higher percentage of students who maintained a normal weight (under the 85th percentile of BMI-for-age) in the intervention group (52.1%) than in the control group (40.7%). Students in the intervention group had also improved academic performance compared to the control group [17].

The “Shape up Somerville” (SUS) is a non-randomized controlled trial conducted over two school years (September 2003–June 2005) in 1178 children in grades 1–3 (average of 8 years old) attending public school in three different communities from Somerville, Massachusetts, United States [18]. This intervention included more physical activity opportunities around the school, such as information on safe routes to school and walking to the school bus; modifications inside the school space, such as new equipment for physical activities; and a dietary intervention. This included taste tests of fruit and vegetables during lunchtime, where children could vote on whether they would like to see those fruits or vegetables on the monthly school menu; new vegetarian recipes and fresh fruit were made available every day for breakfast and lunch; colorful educational posters with nutrition and health information were displayed in the school cafeterias, and food service staff was trained. Additionally, there was an approval of restaurants according to SUS guidelines which offer low-fat dairy products, some dishes in smaller portion sizes, fruits and vegetables as side dishes, and have visible signs highlighting healthier options. After 1 year, results showed that the BMI z-scores were 0.06 lower in the intervention group than in the control group [18]. There was a decrease in overweight and obesity and an increase in remission in both sexes in the intervention group, but the comparison groups were not randomly assigned.

A randomized cluster controlled trial was performed in Mexico on 532 school-aged children from the 2nd and 3rd grades, with an average age of 8.5 ± 0.73 years at baseline (280 children in the intervention and 252 in the control group; each arm with one public and one private school, totaling four) [19]. It aimed to make these children and their parents reduce their sedentary behaviors, consumption of soft drinks and high-fat and salt-containing snack foods and increase their consumption of fruits and vegetables. The intervention consisted of sessions for discussing healthy lifestyles dedicated to the school board and teachers, conducted by nutritionists and physical activity professionals. There were also interactive lessons provided by nutrition graduated students for the children with the intent of increasing their fruit and vegetable intake, physical activity practice, and reducing their intake of soda and high-fat and salt-containing snacks, while simultaneously lowering their TV watching time. There were also nutrition sessions for parents run by nutrition professionals, with the intent of educating them about healthy eating. The results showed that by the sixth month of the intervention, there was a greater decrease in BMI in the intervention group than in the control group (difference of −0.82 kg/m^2^ in children BMI), although this was not sustained in the long-term, after 18 and 24 months [19].

A Multicomponent School Nutrition Policy Initiative on the prevention of overweight and obesity among children was conducted in 1349 students in grades four through six from 10 schools in a US city [20]. This initiative included the following interventions: school self-assessment, in which the schools suggested strategies such as limiting the use of food as reward/punishment, promoting active recess, and serving breakfasts in the classrooms to ensure the students eat a healthy meal; training in nutrition education for the school staff; nutrition education classes for the children; nutrition policies in the intervention schools, such as changing the foods that were sold and served according to the Dietary Guidelines for Americans to meet the nutritional standards; social marketing, such as giving raffle tickets to students who purchased or brought from home healthy snacks and beverages; and parent outreach through nutrition educators in home and school association meetings, report card nights, parent education meetings, and weekly nutrition workshops [20]. The results of this intervention were a 50% reduction in the incidence of overweight. There were significantly fewer children in the intervention schools (7.5%) than in the control schools (14.9%) who had become overweight after 2 years. However, there were no differences in the incidence or prevalence of obesity, nor in the remission of being overweight or obesity after 2 years of follow-up [20].

Donnelly et al. [21] conducted a 2-year trial in students from grades three to five in two school districts in rural Nebraska aiming to reduce obesity and improve physical and metabolic fitness. The intervention consisted of nutrition education, modified school lunches, and increased physical activity. The meals were planned with the kitchen staff according to the Lunchpower! Program. This program consists of energy, fat, and sodium reduced lunches, in agreement with the Healthy People 2000 objectives [22]. According to this, the fat content is restricted to 30% of the total energy intake, the sodium is limited to 1000 mg, the cholesterol to 100 mg, and the dietary fiber is increased to 8 to 10 g per day. There were also nutrition classes given by the teacher, after being trained. These classes included basic nutrition, nutrition for proper growth and development, the relationship between diet and health, healthy food choices, how to reduce fat in the diet, snack alternatives, and food safety. After two years of intervention, the control school showed significantly higher total energy (9%) and total fat (25%). The control school also showed considerably greater values for sodium and smaller for fiber. After the first year of intervention, there were no significant differences between the control and intervention schools in nutrition knowledge. However, after two years of intervention, the intervention school reduced by 45% their wrong answers about nutrition knowledge. Concerning physical activity, the control school practiced significantly more sports outside school compared to the intervention school. After 2 years of the intervention, neither the control nor intervention schools showed significant increases in aerobic capacity. Both schools showed no significant changes in the percentage of body fat, but a significant increase in BMI [21].

The DECIDE-Children Study [23] is a cluster-randomized controlled trial conducted in 1200 Chinese students from four primary schools (8–10 years old). The intervention consisted of health education activities for the parents; supervision and encouragement of the children as a way of increasing their physical activity practice outside of school; school policies to prevent obesity and health education activities for the children. There was also the development of an app called ‘Eat Wisely, Move Happily’ that aids in diffusing information, monitoring the children’s behavior, managing their weight, and giving feedback for the teachers and parents. Since this study is ongoing, the results of this intervention are not yet available [23].

In 2020, a multicenter randomized controlled trial [24] was conducted in 4846 Chinese school children aged 7 to 13 years, in which the intervention consisted of the development of a nutrition handbook that was given to all students; nutrition and health courses to the students, parents, teachers, and health workers about the proportion of the meals, how to choose healthy foods, and how to reduce eating out, unhealthy fast food, sugar-sweetened beverages, and snacks; and displaying informative posters around the school. Courses on physical activity for the parents and physical activity classes for the students were also given. There were no significant improvements in the overall diversity of the food consumption in the intervention group; however, there were some improvements in the diversity of the foods consumed at breakfast and a decrease in the consumption of some unhealthy foods [24]. No effects on children’s BMI were studied.

The Abriendo Caminos Program [25] was implemented in several schools in Illinois, California, Iowa, Texas, and Puerto Rico targeting families of parents and one child aged 6−18 years old (n = 500). This randomized control trial consisted of workshops, presentations and activities on nutrition education, family wellness, and physical activity. There are still no known results from this study.

Another randomized control trial called Healthy Start [26] was conducted in Denmark and targeted school children aged 2 to 6 years (n = 3722) and consisted of guiding families on how to improve their children’s diet and physical activity practices, reduce stress, and improve sleep quantity and quality. Activities included cooking classes, games focused on exercise and motor skills development, and access to a website that provided recipe inspiration and ideas. The clinical effects of this intervention on children’s growth and body composition measures were small [26].

The FIVALIN Project [27] is a quasi-experimental study conducted in 810 children aged 8–12 years and 600 parents in Barcelona. This study consisted of workshops on health education and sports educational sessions. Educational materials, mobile messages to remind parents to attend the workshops, with the date and hour, and videos were sent to families to reinforce the health behaviors encouraged during the workshops and sports educational sessions. This study is ongoing; therefore, there are still no known results.

The CHIRPY DRAGON Intervention [28] was a cluster-randomized controlled trial led in Chinese school children with a mean age of 6.15 years (n = 1641). This school- and family-based obesity prevention program consisted of workshops and family activities to promote physical activity and healthy eating behaviors, and school support to improve physical activity and healthy food provision. After 12 months of intervention, the BMI z- scores of children in the intervention group decreased, along with an increase in the consumption of fruit and vegetables, and a decrease in the consumption of sugar-sweetened beverages and unhealthy snacks. Screen time also decreased and physical activity increased in this group [28].

The Kids in Action [29] was a controlled trial conducted with children aged 9–12 years from four primary schools in Amsterdam. The study consisted of meetings with the children to develop interventions that targeted their physical activity and healthy eating habits. This intervention consists of environmental changes, organizational changes, or educational approaches, and depending on the type of intervention, the executors could be dieticians, sports coaches, or supermarkets in the community. There are no results from this study yet.

In 2018, an education-based intervention study called The ABC of Healthy Eating Project (including 464 students) was conducted in Poland [30]. This study included students aged 11–13 years. The intervention group received a diet and lifestyle-related educational program and both the intervention and the control group partook in school activities with the theme of nutrition and healthy lifestyles. There are still no known results. 

### 3.2. Community-Based Interventions

The MOVE/me *Muevo* was a randomized community trial implemented in 30 recreation centers in San Diego County in a total of 541 families with children between the ages of 5 and 8 years to prevent and control childhood obesity [31]. This program consisted of activities at the recreation centers and participants’ homes, as well as phone calls from health coaches and emailing tip sheets. The intervention families had “Family Health Coaches” who addressed the following nutrition behaviors: increase the consumption of fruit and vegetables through modifications in meal and snack purchases and preparation; decrease the consumption of sugar-sweetened beverages through changes in food purchases and setting limits; increase healthy food portions by modifying the food consumption behaviors; reduce eating out and when eating out, choosing healthier options; increase the availability and accessibility of healthy foods and beverages at home; reduce screen time and avoid eating in front of the television, and increase the number of meals eaten as a family. After 2 years, there were no significant differences between the control and intervention groups concerning BMI or waist circumference [31]. Some changes were observed in the dietary domain, namely a reduction in fat and sugary beverages, which means that it was easier for the participants to adopt healthier behaviors in this field, compared to the more complex and multidimensional attitudes of physical activity [31].

The “Romp & Chomp” is a community-based trial carried out in Australia in children aged 1–5 years old (n = 12,000) and their families [32]. There were changes regarding the provision of water in childcare centers, childcare policies regarding healthy eating and physical activity, and skills in physical activity and nutrition were taught to the childcare professionals. Amongst the nutrition interventions, there were the following: a collaboration with Dental Health Services Victoria, which provided some resources (lunch boxes and drink bottles, and some marketing material for the kindergarten children); training of the staff as a way to support nutrition messages and healthy eating choices for children aged 5 years; support from dental health professionals to the kindergartens, as a way to engage with parents on the topic of healthy eating and with the intent of providing support for the staff to implement health and nutrition policies; access to a dietitian and other allied health professionals through e-mails, phone calls, and site visits; production and distribution of promotional materials (balloons, stickers, posters, postcards). After 3 years of intervention, the 3.5 years old subsample showed considerably lower mean weight, BMI, and z-score BMI, and the 2 and 3.5 years old children showed a considerably lower prevalence of overweight and obesity when compared with baseline values. The intervention group also showed a considerably lower intake of packaged snacks and fruit juice [32].

The Aventuras Para Niños Study is a community-based intervention to promote healthy eating and physical activity and prevent excess weight gain in Latino children [33]. It was performed in thirteen elementary schools, with randomization to assign them to either a family-only intervention, a community-only, or a family+community intervention. In the family-only intervention, professionals would either call the families or make home visits as a way of discussing ways to pass through the difficulties of maintaining a healthy diet and being physically active, by showing them how to prepare healthy meals at home, as well as presenting them with the benefits of encouraging their children to eat healthily and practice physical activity. The community-only intervention included improving the schools’ playgrounds, implementing salad bars, as well as community parks, and displaying water bottles in classrooms for the students. It also included the implementation of better physical education equipment and healthy menus for the children, all of this combined with spreading media messages through posters, news, and point-of-choice messages in grocery stores with healthy messages. The family+community intervention included all of the interventions above. The results showed no noteworthy main effects for the family or community interventions. Therefore, it is possible that not any real effects for the family or community interventions were observed in the BMI z-scores of the children compared with either of those circumstances alone. Despite the lack of significant effects on children’s BMI z-scores, there were several obesity-related behaviors in these children that were changed by the family intervention, such as the increased consumption of fruits and vegetables [33].

The EPODE (Ensemble Prevenons l’Obesité Des Enfants/Together Let’s Prevent Childhood Obesity) aims to reduce childhood obesity through a societal process that consists of childhood settings, local environments, and family norms becoming more supportive and making it easier for children to adopt healthy lifestyles by enjoying healthy eating, active play, and recreation [34]. This program was launched in 2004 in 10 French pilot communities, and targeted children aged 1–12 years, their families, and various local stakeholders who have the power to initiate micro-changes in these children and their families through local initiatives focusing on better and balanced eating habits and the regular practice of physical activity. Recently, there have been some other programs, inspired by the EPODE methodology, such as the Healthy Weight Communities in Scotland or the JOGG program in the Netherlands.

The Pacific Obesity Prevention in Communities (OPIC) Project was carried out in four countries, Australia, Fiji, New Zealand, and Tonga, over 30 months, between 2004 and 2009 [35]. This was a complex community-based intervention that included 18,000 secondary-school children (aged 12–18 years) from eight ethnic and cultural groups, 60 multi-professional research staff, 300 stakeholders and partner organizations, and 27 higher degree research students. The interventions varied across sites, but all sites included targeting reductions in the consumption of high-sugar content drinks and energy-dense snacks and increasing physical activity. The authors state that the project may have positive effects on diet and physical activity, but the effects on childhood obesity are not clearly described [36].

### 3.3. Interventions through Mass Media

Some interventions to tackle childhood obesity through mass media have been based on restrictions on food advertising to children. It has been shown that restricting the number of hours spent watching television (TV) can be an effective approach to reduce the prevalence of childhood obesity, and reducing the meals in front of a TV has been shown to be as important as increasing physical activity [37]. Energy-dense foods and drinks and fast-food companies often target children in their advertisements, since they are very easily influenced at young ages, namely through TV commercials. Thus, reducing the time spent in front of the TV might be a useful strategy to try to reduce the childhood obesity prevalence. Sweden has banned TV commercials/advertisements to children under 12 and TV advertising to children. Norway, Denmark, Austria, Ireland, Australia, and Greece have also imposed some restrictions on advertising to children [38], as well as Portugal [39]. 

### 3.4. Food Sector Interventions

Food taxation is a primordial prevention measure taken that is currently being applied in several countries, such as some parts of the USA and Canada [40], to reduce the intake of unhealthy foods and, in the long term, their health effects such as obesity. Some examples are high-volume foods with low nutritional value, such as soft drinks, confectionery, and snack foods. Portugal has also adopted the taxation of sugar-sweetened beverages as an intervention to reduce its high consumption in the country [41]. There was a decrease of 6.58 million liters of sugar-sweetened beverages sold per year, which translates into a decrease in consumption of 21% compared to the baseline consumption data from the National Dietary Survey [41]. The number of cases of obesity prevented by taxing sugar-sweetened beverages was studied, concluding that there was a higher impact on adolescents (0.012%), preventing 0.76 cases of obesity yearly [41]. 

According to Teng et al. studies suggest that the implementation of sugar-sweetened beverages taxes worldwide has proven effective in reducing sugar-sweetened beverages purchases and intake [42]. Evidence also shows that the taxation of sugar-sweetened beverages might be an effective tool to reduce the consumption of sugar-sweetened beverages and an important component to prevent obesity [42]. Roberts et al. suggest that a fiscal strategy could very likely reduce the purchase of high-sugar content products, even if in the short term [43].

Another measure currently being taken is the addition of logos or some type of labeling to alert the consumers to the healthier products, making it easier for them to choose healthy foods. Although it is not directly focused on childhood obesity, it may have indirect effects. Anastasiou et al. reported that food labeling may affect the consumer’s dietary intake; however, results are inconclusive [44]. It is uncertain if using health-related claims is beneficial or damaging. Nonetheless, other than health-related claims, negative effects derived from food labeling seem highly unlikely according to the evidence. Therefore, food labeling should continue to be promoted in policies and education programs [44].

An example of this intervention is the “Pick the Trick” Program, conducted in Australia and New Zealand, providing foods with symbols for the consumers that make it easier to identify the healthier choices [45]. In Europe, the WHO European Food and Nutrition Action Plan 2015–2020 identifies the introduction of interpretative, consumer-friendly labeling on the front of packages as a priority policy issue [46]. Although the majority of countries in the region (n = 15) have some form of front-of-pack labeling, fewer countries have interpretive systems which provide judgments about the relative healthfulness of foods. Among other future policies, there is the intention of the application of a single front-of-pack labeling system in all countries. A WHO report summarizes the existing evidence on the development processes and effectiveness of front-of-pack food labeling policies in the WHO European region [47].

The portion sizes have also been getting increasingly larger over the past four decades in most high-income countries [48,49]. Despite this increase in portion sizes, few countries report measures to reduce them. Most measures are focused on information to consumers rather than changes in the food and drink environment [50].

**Table 1:** Summary of dietary interventions on childhood obesity and their main characteristics and results.

Author (Study Title) (Reference)	Country, Year	Type of Intervention	Intervention Description	Target Audience	Results
**School-Based Interventions**					
Niederer I, et al. (Ballabeina study) [13]	Switzerland, 2009	Cluster randomized controlled single-blinded trial	Lessons on nutrition (balanced nutrition and healthy nutritional behaviors in a didactic way), physical activity program, media use, and sleep, and adaptation of the built environment of the preschool.	Preschool children (mean 5.1 years) (n = 652), the parents, and the teachers	No differences in children’s BMI were found between groups. However, the intervention group had a reduction in body fat percentage, better motor agility, as well as benefits in reported physical activity, media use, and eating habits.
Singh AS, et al. [14,15]	Netherlands, 2006	School-based trial	Educational component (classes in biology and physical education, and a computer-based information program); and an environmental component (e.g., serving smaller portion sizes in the canteen and healthier options, restricting access to vending machines, and food awareness by posters).	Students from the ages of 12–14 years (n = 1108)	With a twelve-month follow-up, a reduction in the skinfold thickness of the intervention groups was found, as well as lower consumption of sugar-containing beverages, and less screen time (but only in boys).
Kain J, et al. [16]	Chile, 2004	School-based obesity-prevention trial	Weekly classes on physical activity and healthy nutrition for parents and students. Some environmental changes were also made (e.g., school kiosks were instructed to offer healthier choices and at the same time remain lucrative).	Parents and students from 1st to 8th grade; 2141 schools in the intervention group and 945 in the control group.	After 6 months, there was a reduction of body mass index (BMI) z-scores in boys and better physical fitness in both genders. On the other hand, the modifications in the kiosk’s food availability did not seem to change the students’ food choices.
Hollar D, et al. (Healthier Options for Public Schoolchildren (HOPS)) [17]	Florida, US, 2004–2006	Randomized trial	Modifications in the school menu, school gardens, and physical activity; monthly newsletters with healthy nutrition and physical activity lessons for the students and parents.	6 elementary schools (4588 children aged 6 to 13 years; 48% Hispanic)	After 2 years, a higher percentage of students who maintained a normal weight (<85th percentile of BMI-for-age) was found in the intervention group (52.1%) when comparing with the control group (40.7%). Students in the intervention group had improved academic performance.
Economos CD, et al. (Shape up Somerville) [18]	United States, (September 2003–June 2005)	Non-randomized controlled trial	Dietary intervention (e.g., promotion of fresh fruit and vegetables and taste tests, posters with nutritional and health information, training of food staff, modification of food offers in restaurants according to the study guidelines); increase of physical activity opportunities around the school (e.g., information on safe routes); modifications inside the school space (e.g., new equipment).	1178 children (average 7.92 years) attending public school in three different communities from Somerville, Massachusetts	After 1 year, the BMI z-scores were 0.06 lower in the intervention group than in the control group. There was a decrease in overweight and obesity and an increase in remission in both sexes in the intervention group. The study design did not include randomization of the intervention.
Bacardí-Gascon M, et al. [19]	Mexico, 2012	Randomized cluster controlled trial	Sessions discussing healthy lifestyles to the school board and the teachers; interactive lessons to the children to increase fruit and vegetables intake and physical activity practice, and reduce soda and high fat and salt-containing snacks intake, while simultaneously decreasing TV watching time; healthy eating sessions to parents.	532 school-aged children from 2nd and 3rd grade	By the sixth month, there was a greater decrease in BMI in the intervention group than in the control group (difference of −0.82 kg/m^2^ in children BMI), although it was not sustained after 18 and 24 months of intervention.
Foster GD, et al. [20]	USA, 2008	Multicomponent School Nutrition Policy Initiative	School self-assessment (e.g., strategies like limiting the use of food as reward/punishment, promoting active recess, and serving breakfasts in classrooms); training of school staff and children in nutrition education; nutrition policies (e.g., changing sold foods); social marketing; school association meetings/workshops.	1349 students in grades 4 through 6 from 10 schools	There were significantly fewer children in the intervention schools (7.5%) than in the control schools (14.9%) who became overweight after 2 years, but no differences after 2 years of follow-up.
Donnelly JE, et al. [21]	Nebraska, USA, 1996	2-year trial	Nutrition education (basic nutrition, diet, and general health, nutrition for growth and development, healthy food choices, snack alternatives, food safety), modified school lunches (meals planned according to the Lunchpower! Program aiming to reduce energy, fat, and sodium lunches), and increased physical activity.	Students from grades 3 to 5 in two school districts in rural Nebraska (n = 338)	After 2 years of the intervention, both schools showed no significant changes in the body fat percentage, but a significant increase in the BMI. The control school showed significantly higher total energy, total fat and sodium intake, and lower fiber intake.
Liu Z, et al. (The DECIDE-Children study) [23]	China, 2019	Cluster-randomized controlled trial	Health education activities for parents and children; supervision and encouragement of children’s physical activity practice outside of school; school policies to prevent obesity. Development of an app called ‘Eat Wisely, Move Happily’ that aids in diffusing information, monitoring the children’s behavior, and managing their weight.	4-grade primary schools (8–10 years old) (n = 1200)	No known results.
Xu H, et al. [24]	China, 2020	Multicenter randomized controlled trial	Development of a nutrition handbook that was given to all students; nutrition and health courses to students, parents, teachers, and health workers (e.g., meals proportion, how to choose healthy foods, reduce eating out and unhealthy foods); informative posters around the school; course on physical activity for parents, and physical activity classes for students.	4846 school children aged 7–13 years	The effects on children’s BMI were studied. There were some improvements in the diversity of the foods consumed at breakfast and a decrease in the consumption of some unhealthy foods.
Hannon BA, et al. (*Abriendo Caminos* Program) [25]	Illinois, California, Iowa, Texas, and Puerto Rico, 2019	Randomized control trial	Workshop presentations and activities on nutrition education, family wellness, and physical activity.	Families of parents and 1 child aged 6–18 years (n = 500)	No known results.
Olsen NJ, et al. (Healthy Start) [26]	Denmark, 2020	Randomized controlled trial	Guidance on how to improve the child’s diet and physical activity, quantity and quality of sleep, and reduce their stress. Cooking classes, games focused on exercise and motor skills development, access to a website with recipes.	Children aged 2 to 6 years (n = 3722)	The clinical effects of this intervention in the children’s growth and body composition were small.
Homs C, et al. (FIVALIN project) [27]	Barcelona, 2021	Quasi-experimental design	Workshops on health education and sports educational sessions.	810 children aged 8–12 years and 600 parents	No known results.
Li B, et al. (The CHIRPY DRAGON intervention) [28]	China, 2019	Cluster-randomized controlled trial	Workshops and family activities to promote physical activity and healthy eating behaviors; school support to improve physical activity and healthy food provision.	School children with a mean age of 6.15 years (n = 1641)	There was a decrease in the BMI z-scores of the children in the intervention group, along with an increase in the consumption of fruit and vegetables, and a decrease in the consumption of sugar-sweetened beverages and unhealthy snacks. There was also a decrease in screen time and an increase in physical activity in this group.
Anselma M, et al. (Kids in Action) [29]	Amsterdam, 2019	Controlled trial	Meetings with children to develop interventions that targeted their physical activity and healthy eating habits. These interventions consist of environmental changes, organizational changes, or educational approaches.	Children aged 9–12 years from four primary schools	No known results.
Hamulka J, et al. (The ABC of Healthy Eating Project) [30]	Poland, 2018	Education-based intervention study	Diet and lifestyle-related programs for the intervention group and school activities with the theme of nutrition and healthy lifestyles for both the intervention and the control group.	School children aged 11–13 years. (464 students)	No known results.
**Community-based interventions**					
Elder JP, et al. (MOVE/me Muevo) [31]	San Diego County, USA, 2014	Randomized community trial	Activities and phone calls from health coaches on how to increase the consumption of fruit and vegetables; decrease the consumption of sugar-sweetened beverages; increase healthy food portions; reduce eating out and do healthier options when eating out; increase the availability and accessibility of healthy foods and beverages at home; reduce the screen time and avoid eating in front of the TV, and increase the number of family meals.	541 families with children between the ages of 5 and 8 years old	After 2 years, there were no significant differences between the control and the intervention group concerning BMI or waist circumference. Some changes were observed in dietary intake, namely a reduction in fat and sugary beverages in the intervention group.
De Silva-Sanigorski A, et al. (Romp & Chomp) [32]	Australia, 2020	Community-based trial	Changes in the provision of water in childcare centers, childcare policies regarding healthy eating and physical activity; teaching of skills in physical activity and nutrition to the childcare professionals; production and distribution of promotional materials (balloons, stickers, posters, postcards).	Children aged 1–5 y (n = 12,000) and their families	After 3 years of intervention, the 3.5 years old subsample showed considerably lower mean weight, BMI, and z-score BMI, and the 2 and 3.5 years old children showed a considerably lower prevalence of overweight and obesity when compared with the baseline values. The intervention group also showed a considerably lower intake of packaged snacks and fruit juice.
Crespo NC, et al. (The Aventuras Para Niños Study) [33]	Southern California, 2003	Randomized Community-based trial	Three arms: family-only, community-only, or family+community intervention. In the family-only intervention, professionals call/make home visits to discuss how to maintain a healthy diet, prepare meals, and be physically active. The community-only intervention included improving the school’s playgrounds, implementing salad bars, as well as community parks, displaying water bottles in the classrooms for the students, better physical education equipment and healthy menus for the children, all of this combined with spreading media messages through posters, news and point-of-choice messages in grocery stores, with health messages. The family+community included all described.	811 predominantly Mexican immigrant/Mexican-American mothers with children in kindergarten through second grade	No noteworthy main effects nor interactions for the family or community interventions were found, including on BMI z-scores. Despite the lack of significant effects on the children’s BMI z-score, there were multiple obesity-related behaviors in these children that were changed by the family intervention, like increased consumption of fruit and vegetables.
Borys JM, et al. (EPODE (Ensemble Prevenons l’Obesité Des Enfants/Together Let’s Prevent Childhood Obesity) [34]	France, 2004	Community-based intervention	Changes in local environments, childhood settings, and family norms to make them more supportive and aid the adoption of healthy lifestyles in children.	Children aged 1–12 years, and their families, as well as a wide variety of local stakeholders in 10 French pilot communities	No known results.
Swinburn BA, et al. and Schultz JT, et al. (Pacific Obesity Prevention in Communities (OPIC) project) [35,36]	Australia, Fiji, New Zealand, and Tonga, over 30 months, between 2004 and 2009	Community-based intervention	Interventions aiming to reduce the consumption of high sugar content drinks and energy-dense snacks and increase physical activity.	18 000 children 12–18 years, 300 stakeholders, 60 multi-professional research staff, 27 research students.	The authors state that the project can produce positive effects in diet and physical activity, but effects on childhood obesity are not clearly described.
**Interventions through mass media**					
World Health Organization and Assembly of the Republic (TV ban/restrictions of food commercials to kids in several countries [38] and Portugal) [39]	Sweden, Norway, Denmark, Austria, Ireland, Australia, Greece, and Portugal, 2019	Mass-media based- intervention	Sweden has banned TV food commercials for children under the age of 12 and TV food advertising for children. Norway, Denmark, Austria, Ireland, Australia, and Greece have also made some restrictions on commercials for children. Portugal approved a law to restrict advertising to children for foodstuffs and beverages of high energy value, salt, sugar, and saturated fatty acids content.	Children	No efficacy results. However, energy-dense foods and drinks and fast-food companies often target children in their advertisements, since they are very easily influenced at this age, namely through TV commercials.
**Interventions through the Food Sector**					
Goiana-da-Silva F, et al. (Taxation of sugar-sweetened beverages) [41]	Portugal, 2017	Food sector intervention	Taxation of sugar-sweetened beverages as an intervention to reduce its high consumption in the country.	Community	Decrease of 6.58 million liters per year, which translates into a decrease in consumption of 21% compared to the baseline consumption data of IAN-AF 2015–2016. The number of cases of obesity prevented had a higher impact in adolescents (0.012%), preventing 0.76 cases of obesity yearly, followed by an impact of 0.062% in adults aged 18 to <65 years, and the children showed an impact of 0.049%. These data show that Portugal achieved its goal, decreasing sales of sugar-sweetened beverages.
Young L, et al. (“Pick the Trick” program) [45]	Australia and New Zealand	Food sector intervention	Providing foods with symbols for the consumers making it easier to identify the healthier choices.	Community	No known results
Kelly B, et al. and Nielsen S, et al. (WHO front-of-pack labeling system) [47]	WHO-E Food and Nutrition Action Plan 2015–2020	Food sector intervention	Among other future policies, there is the intention of application of a single front-of-pack labeling system in all countries.	Community	No known results

## 4. Discussion

This study aimed to review the most recent literature on dietary interventions for the prevention of childhood obesity. It describes different levels of interventions: the school level, the community level, the mass media, and the food sector level, and provides an overview of their effectiveness (the ability to show consistent results overtime on decreasing children’s BMI), which stand out from previous reviews.

Given the complexity and multifactorial nature of obesity, it is consensual that there is a need to take actions at multidimensional levels, including individual, familial, institutional, and environmental. The majority of the studies included in this review aiming to reduce/manage childhood obesity were school-based interventions, with some addressing the whole community, and some including distal interventions through the food sector and mass media, which may have an indirect effect on childhood obesity by changing food behaviors. 

Children are highly influenced by social and environmental conditions, so at these ages, the modification of the environment is expected to play an important role. However, most of the programs/interventions conducted focus mainly on person-based educational approaches, such as nutrition/diet education sessions combined with the promotion of physical activity and lifestyles to students, parents, and school staff, and less on environmental changes that facilitate healthier behavioral choices. Only some trials [13,14,16,17,18,20,21,30,31] have focused on capacity building and macro-policy changes, such as the adaptation of the built environment of the school, serving smaller portion sizes and increasing the availability and accessibility of healthy foods and water in schools, and restricting access to vending machines, for example. 

Multidimensional intervention studies are usually difficult to evaluate and highly depend on the complexity of evaluation designs (e.g., only outcome evaluation vs. complex evaluation including process, impact, and outcome). Moreover, especially in the multidimensional community-based programs, it is hard to distinguish which part of the intervention was the most effective.

Overall, most of the intervention studies did not show consistent effects on changing children’s BMI. A large number of studies, mainly based on school interventions, did not show very effective results, which may be a reflection of the difficulties experienced trying to obtain significant results when relying only on school-based interventions. In fact, the small weight reductions described in most studies could be clinically irrelevant. It is difficult to figure why the interventions taken until now to prevent/reduce childhood obesity have failed to provide substantial results in terms of effectiveness. The ineffectiveness of some interventions may be due to insubstantial evaluation, or because studies were too short to detect appropriate outcomes, or, simply, because they do not work [51]. Another possible explanation is the lack of interventions at multiple levels of determinants, especially environmental changes (distal level). Importantly, little is known about the sustainability of interventions over time [51]. However, other positive results, such as the change of dietary behaviors or physical activity performance have been described and should not be discarded. 

Actions to prevent childhood obesity need to be taken in multiple settings and incorporate a variety of approaches and involve a wide range of stakeholders [51]. Complex interventions focused on environmental changes and the strengthening of individuals and communities as well as macro-policy changes seem to be promising strategies to reduce childhood obesity without increasing socioeconomic inequalities [52]. The best approach should include the family context and contemplate early life determinants. An approach that could be much more effective to prevent obesity is a combination of interventions that promote healthier diets and increase physical activities through society, rather than an approach focused solely on school environments [52]. Focusing on mass media campaigns and political actions to prevent obesity by influencing people’s eating choices and the increase of physical activities might be an effective approach to this problem [52].

Overall, sustained interventions are likely to be required at several levels, at an individual level in schools and community settings to effect behavioral change, and in sector changes involving different stakeholders [51].

## 5. Conclusions

Most dietary interventions to tackle childhood obesity focus mainly on person-based educational approaches and less on environmental changes to offer healthier behavioral choices. Most of them failed to reduce childhood obesity.

The creation of environments supportive of healthier behaviors seems to be the best approach to mitigate the challenge that is childhood obesity. Complex and multilevel interventions focused on environmental changes and the strengthening of individuals and communities, including family, as well as macro-policy changes will have the potential to tackle childhood obesity without increasing socioeconomic inequalities.

## Data Availability

Not applicable.
